# Is There Any Difference in the Outcome of Geriatric and Non-Geriatric Severely Injured Patients?—A Seven-Year, Retrospective, Observational Cohort Study with Matched-Pair Analysis

**DOI:** 10.3390/jcm9113544

**Published:** 2020-11-03

**Authors:** Kai O. Jensen, Maximilian Lempert, Kai Sprengel, Hans P. Simmen, Carina Pothmann, Mathias Schlögl, Heike A. Bischoff-Ferrari, Christian Hierholzer, Hans C. Pape, Valentin Neuhaus

**Affiliations:** 1 Department of Trauma, University Hospital Zurich, University of Zurich, 8091 Zurich, Switzerland; maximilian.Lempert@usz.ch (M.L.); kai.sprengel@usz.ch (K.S.); hanspeter.simmen@usz.ch (H.P.S.); carina.pothmann@usz.ch (C.P.); christian.hierholzer@usz.ch (C.H.); hans-christoph.pape@usz.ch (H.C.P.); valentin.neuhaus@usz.ch (V.N.); 2 University Clinic for Acute Geriatric Care, City Hospital Waid, 8037 Zurich, Switzerland; mathias.schloegl@waid.zuerich.ch; 3 Department of Geriatrics and Ageing Research, University Hospital Zurich, University of Zurich, 8091 Zurich, Switzerland; heike.bischoff@usz.ch

**Keywords:** outcome, geriatric trauma, polytrauma, matched pair analysis

## Abstract

Geriatric trauma is expected to increase due to the lifestyle and activity of the aging population and will be among the major future challenges in health care. Therefore, the aim of this study was to investigate differences between polytraumatized geriatric and non-geriatric patients regarding mortality, length-of-stay and complications with a matched pair analysis. We included patients older than 17 years with an Injury Severity Score (ISS) of 16 or more admitted to our level 1 trauma center between January 2008 and December 2015. The cohort was stratified into two groups (age < 70 and ≥ 70 years). One-to-one matching was performed based on gender, ISS, mechanism of injury (penetrating/blunt), Glasgow coma scale (GCS), base excess, and the presence of coagulopathy (international normalized ratio (INR) ≥ 1.4). Outcome was compared using the paired *t*-test and McNemar-test. A total of 1457 patients were identified. There were 1022 male (70%) and 435 female patients. Three hundred and sixty-four patients (24%) were older than 70 years. Matching resulted in 57 pairs. Mortality as well as length-of-stay were comparable between geriatric and non-geriatric polytraumatized patients. Complication rate (34% vs. 56%, *p* = 0.031) was significantly higher in geriatric patients. This indicates the possibility of similar outcomes in geriatric polytraumatized patients receiving optimal care.

## 1. Introduction

Demographic changes are ubiquitous and cause a growing number of elderly patients. In Switzerland, we expect an increase in people aged 65 years and above from 29.1% of the population in 2015 up to 48.1% in 2045 [1]. A similar increase can be expected for most of Europe [2]. Recent studies report that patients over 65 years already account for 23% of all trauma admissions and that trauma is the fifth leading cause of death in this group of the population [3,4].

Despite its growing importance in medicine, geriatric trauma has not been defined in a universally accepted way. Some clinicians use an age threshold as an accepted distinction. The definition of what might be geriatric age, however, ranges from 60 up to 75 years of age [5,6,7,8].

Furthermore, elderly people are living an increasingly independent and active lifestyle [9]. As a consequence, a growing number of elderly patients sustain severe injuries in unintentional incidents [10,11,12,13]. This poses a challenge in trauma care, since advanced age alone is regarded as a risk factor for adverse outcomes in trauma [9,14]. Champion et al. reported in 1990 a three times higher mortality after trauma in older patients (> 70 years) compared to persons less than 55 years of age [15]. Mortality was found to be six-fold higher compared to younger patients with the same degree of injury [16]. Elderly patients have reduced physiological resources making them more prone to complications regardless of the injury or comorbidities, thus limiting their potential to respond to trauma, shock, and hypoxia [17,18,19]. Recent studies have shown that in the elderly patient with severe injuries, age and Injury Severity Score (ISS) are independently linked to mortality [20,21,22]. Furthermore, concomitant diseases also contribute to mortality in the elderly, but the effects seem to fade away with an increasing ISS [23,24].

Due to the current lack in the literature, the aim of our study was to determine whether there are differences between geriatric and non-geriatric patients in (1) mortality, (2) length of stay, and (3) complications due to age in a matched-pair analysis of severely injured patients.

## 2. Methods

In this retrospective database study, we analyzed all polytraumatized patients (orthopedic and non-orthopedic) admitted to a Level-1-trauma center between January 2008 and December 2015.

### 2.1. Patients

The inclusion criteria were all adult trauma patients (older than 17 years) with an ISS, coded by a trained study nurse, of 16 or more to evaluate severely injured patients [25], who were primarily or secondarily admitted to our Level-I trauma center. Exclusion criteria were missing data. Data were prospectively collected—in accordance with the German Trauma Registry DGU^®^—in four consecutive time phases from the site of the injurious incident until discharge from hospital: (A) pre-hospital phase, (B) emergency room and initial surgery, (C) intensive care unit, and (D) discharge. The documentation includes detailed information about demographics, injury pattern, comorbidities, pre- and in-hospital management, clinical course on intensive care unit, relevant laboratory findings including data on transfusion, and outcome of each individual. More specifically, the following parameters were collected: patient demographics (age, gender, trauma mechanism), Glasgow coma scale (GCS) [26] at admission, abbreviated injury score (AIS) [27] for each region, ISS [27], American Society of Anesthesiologists-Score (ASA-score) [28], hemodynamic parameters (systolic blood pressure (SBP) and pulse rate (PR) at admission), laboratory values (serum hemoglobin levels, serum hematocrit, lactate levels, base excess levels, thrombocyte counts, international normalized ratio (INR)) at admission, and comorbidities. Professional medical coders coded the injuries, diseases, and the procedures. Complications were defined in the presence of the corresponding International Classification of Diseases (ICD-10) code of an acute disease, such as acute hemorrhagic anemia (hemoglobin < 117 g/L), sepsis (Systemic inflammatory response syndrome (SIRS) criteria [29] (SIRS = two or more of the following criteria being met: temperature ≥38.0 °C or ≤36.0 °C, tachycardia with heart rate ≥ 90/min, respiratory rate ≥ 20/min or PaCO2 ≤ 4.3 kPa or 33 mmHg, leukocytes > 20,000/mm^3^ or ≤ 4000/mm^3^) and any positive microbiological sample), urinary tract infection (positive microbiological urin sample), respiratory failure (need for intubation) as well as pneumonia (positive microbiological sample and radiological findings), acute renal failure (RIFLE criteria [30]), pulmonary embolus (radiological (ct) findings), deep vein thrombosis (detection in ultrasound), myocardial infarction (Electro cardiographie(ECG) changes or laboratory findings), surgical site infection (laboratory and clinical findings), wound dehiscence, and delirium.

### 2.2. Outcome

Primary outcome parameters were (1) in-hospital mortality, (2) length of hospital stay (LOS), and (3) occurrence of the complications mentioned above.

### 2.3. Case-Matched Design

We identified 1464 consecutive polytraumatized patients. In order to establish a matched-pair analysis the cohort was stratified into two age groups. One group consisted of patients aged less than 70 years (“non-geriatric”, *n* = 1117), the other group of patients equal or older to 70 years (“geriatric”, *n* = 346). The age border 70 years was chosen due to in-hospital protocols and the associated geriatric co-management. Next, one-to-one pairing was performed by the statistical software program based on gender, exact ISS, mechanism of injury (penetrating/blunt), exact GCS at admission, base excess (tolerated variance ± 1), and the presence of coagulopathy (dichotomized; defined as an INR ≥1.4). These are the most frequently used covariates in trauma mortality studies [31]. This matching will allow the comparison of the effect of age on outcome after following trauma.

### 2.4. Statistical Analysis

Additional statistical analysis was performed using SPSS^®^ for Windows 24.0 (SPSS, Chicago, IL, USA). Outcome as well as baseline values were compared between groups. The matched-pair analysis was performed using a paired *t*-test and McNemar-test. Data were presented as the mean ± standard deviation for continuous and as absolute and relative numbers for categorical factors. *p*-values less than 0.05 were considered significant.

### 2.5. Ethics Approval and Consent to Participate

The institutional review board (IRB) and cantonal ethical review board approved the study (KEK-ZH-No. 2011-0382, PB_2016_01888), and this study was performed in accordance with the ethical standards laid down in the 1964 Declaration of Helsinki and its later amendments.

## 3. Results

For this study, 1457 patients with a mean age of 62 (±21) years were identified (Table 1). A total of 1022 male (72%) and 435 female patients were included with 346 patients aged 70 years or older. The mean ISS in the entire cohort was calculated as 32 points (±17), following 95.1% blunt trauma mechanism with a GCS of 9.5 (±5.8) points at admission. The most frequently diagnosed injuries in geriatric (G), respectively, in non-geriatric (NG) trauma patients were traumatic brain injuries (G 89.5%; NG 78.9%), thoracic (G 62.3%; NG 60.8%),extremity injuries (G 49.0%; NG 50.0%), as well as abdominal injuries (G 19.3%; NG 23.2%). The initial base excess after matching was −2.6 (±2.6). Coagulopathy after matching was present in 8.9%.

Matching resulted in 57 pairs (Table 2). The mean age of the non-geriatric trauma patients was 47 (±16) years in contrast to 79 (±6.3) years (*p* < 0.001) in the geriatric trauma cohort.

**Figure 1:**
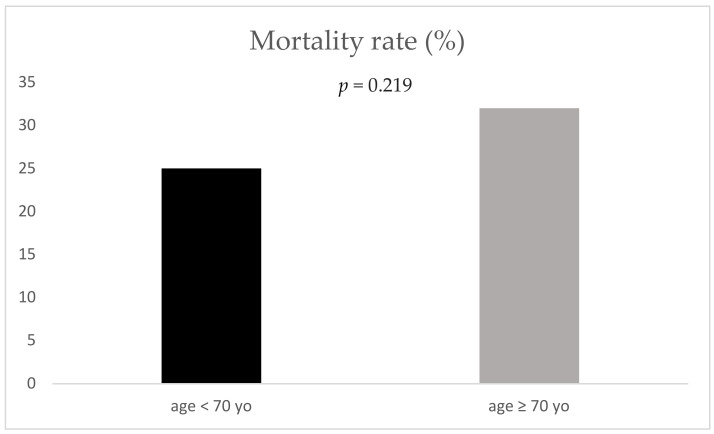
Mortality rate for the two age groups; yo: years old.

**Figure 2:**
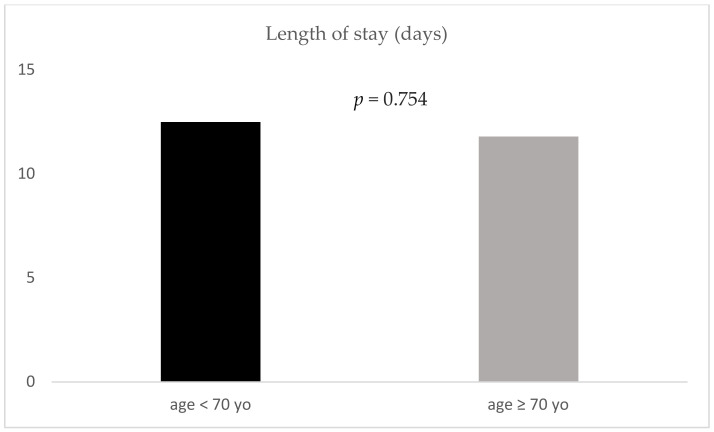
Length of stay for the two age groups; yo: years old.

Mortality (25% vs. 32% respectively; *p* = 0.219) as well as length of stay (12.5 (±13.3) days vs. 11.8 (±11.8) days respectively, *p* = 0.754) demonstrated no significant difference between geriatric and non-geriatric patients (Figure 1 and Figure 2).

**Figure 3:**
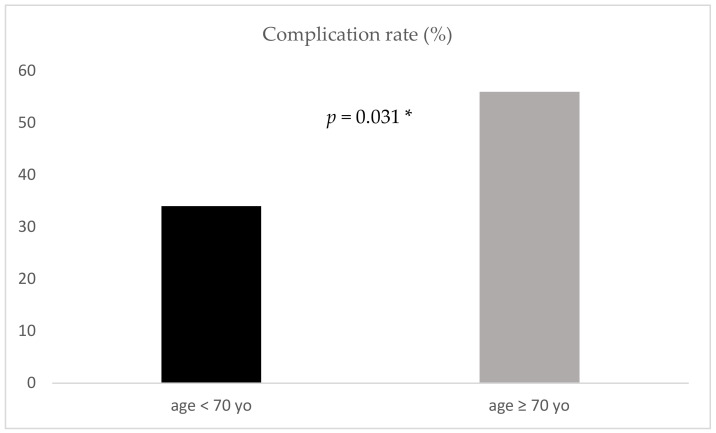
Complication rate for the two age groups; yo: years old; *: significant.

**Figure 4:**
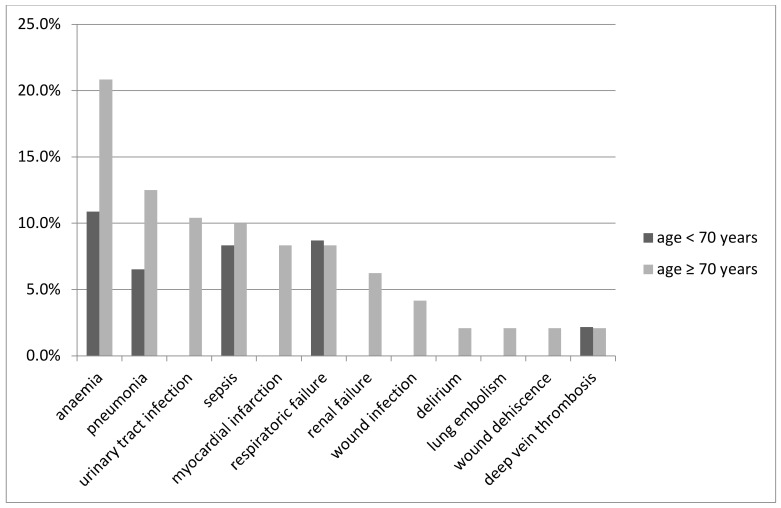
Type of complication for the two age groups.

Patients were predominately discharged to rehabilitation (G 29.8%; NG 36.8%), back home (G 26.3%; NG 19.3%), or to another hospital (G 26.3%; NG 19.3%). Only the complication rate was significantly increased for geriatric patients (34% vs. 56%, *p* = 0.031) (Figure 3). The most common complications in our cohort are shown in Figure 4.

## 4. Discussion

The aim of our study was to investigate the differences in the outcome of elderly polytraumatized patients compared to young patients with the same gender, ISS, mechanism of injury, GCS at admission, base excess, and presence of coagulopathy.

Our main results after matching are as follows:

1. Mortality demonstrated no significant difference between geriatric and non-geriatric patients.
2. Length of stay demonstrated no significant difference between geriatric and non-geriatric patients.
3. Geriatric trauma patients had a significantly higher rate of complications.

### 4.1. Strengths/Limitations

The main strength of this study is the strict matching criteria which are all proven and validated predictors of outcome in trauma [20,32,33,34,35]. With this study concept, we were able to study the effect of age on the outcome of severely injured patients.

The strongest limitation of this study lies within its nature as a retrospective register study. The available data are limited, and missing data cannot be accessed. Data concerning comorbidities, cognitive and motoric status/disabilities, or pre-existing medications are scarce and might change the results. The follow up is limited to the in-hospital results. Therefore, we cannot make any statements regarding long term mortality or morbidity. Furthermore, we could not match the pairs concerning comorbidities as they differed too much, or information was not sufficient.

### 4.2. Mortality

There was a trend towards increased mortality in the older group compared to the younger group, but the difference was not statistically significant. These results are in contrast to recent studies [11,13,23]. However, these studies did not use matched-pair analysis.

With the significantly increased rate of complications and additional predisposing negative factors for survival, one would expect a significantly higher mortality rate in the geriatric group. Possible reasons for this discrepancy may firstly be a higher rate of very early death in the older population, meaning that the most severely injured and vulnerable old patients died prior to hospital allocation or died in the resuscitation area in the emergency department, which led to a lack of data and, therefore, are not included in our data set. On the other hand, the rate of death in the consecutive medical course following hospital treatment is also higher in the elderly than in younger groups, possibly pointing to a larger portion of deaths of geriatric patients a short-time after discharge, which are also not recorded in our study [13,36,37].

Furthermore, some of the matching criteria require critical discussion. Even though GCS, coagulopathy, Base Excess(BE), and ISS are considered to be strong predictors for adverse outcome in trauma [31], elderly patients present with more comorbidities, hence there might be a bias within the matching itself due to pre-existing conditions [20,32,33,34,35]. It is more likely that a pre-existing decreased GCS would be found in an older patient. The same may apply for the base excess. In contrast to a young patient, a pathological base excess in an old patient can be the consequence of a chronic disease. Even the coagulopathy may result from pre-existing medication. All these factors may lead to the matching of pairs with different severities of trauma resulting in the same ISS with consecutive differences in mortality. However, the results of the mortality rate amongst the older patients may also be considered very encouraging as it demonstrates the potential for recovery after severe injuries even in the older population.

The older patients overall stayed for a shorter time at the trauma unit than the patients in the younger group. This might be surprising, as they developed more post-traumatic complications than their younger counterparts. However, with older people more often living in (medical) facilities, some of them can be discharged earlier and in less favourable physical states because they receive medical care at home. Likewise, early hospital-intern transfer to an acute geriatric rehabilitation clinic after the acute phase from the trauma unit counts as discharge in the database.

### 4.3. Complications

We found a significantly higher rate of complications in the group of older patients compared to the younger ones after matching. This is consistent with the results of other groups [13,18,36,38]. Providing trauma care to geriatric patients is particularly challenging as these patients present with more comorbidities and smaller physiological reserves rendering them more susceptible to developing complications [36,39,40], a clinical state that is defined as frailty [9,41]. A frail individual is more vulnerable to developing negative health related events when exposed to a stressor [17,19,41,42]. Frailty is not only found in the elderly, but its rate increases exponentially with age [43].

Typical complications were found in the group of elderly patients. First, the higher rates of anaemia might be multifactorial and partly be caused by the fact that anaemia in general is a more common pre-existing state in the elderly population rising up to 40% due to underlying diseases [44]. Second, we found elderly patients to have a significantly higher risk of developing an acute coronary syndrome, which matches the results of other groups even without the presence of trauma [45]. The additional factors of anaemia, due to blood loss, and the physiological stress of trauma might even further increase that risk. Unless the diagnosis of a myocardial infarction is made either by ECG changes or laboratory findings during the clinical course, there might be a slight overestimation of this complication when the patient suffers from a heart attack directly before the admittance. Third, another complication mainly amongst the older polytrauma patients was pneumonia. Due to physiological changes, comorbidities, and longer periods of immobilization in the elderly, they are generally more prone to pneumonia than their younger counterparts [40,46,47,48].

Another explanation for the higher rate of complications in older patients can also be found in the possible lack of diagnosis at the time of admission [40]. Older people are commonly admitted with a multitude of comorbidities requiring different types of medical treatment. Yet, after a major trauma, medical history might not be available at the point of admission, leading to pre-existing chronic diseases not being properly treated or to pharmaceutical interactions between the patient’s standard and administered emergency-medications further aggravating the patient’s physiological situation.

A very surprising result of our study was the low rate of delirium in elderly patients. Whereas de Vries et al. reported a delirium rate of 20.1% amongst elderly trauma patients [11], we only observed delirium in 2.1% of old patients. This finding bias might partly be explained by the incomplete application of screening tools to detect delirium in our cohort. At that time, a standardized screening method was lacking for diagnosing a delirium. Currently, all geriatric patients are screened for delirium and co-treated by geriatrics in our geriatric trauma centre.

## 5. Conclusions

With this matched pair analysis, we were able to demonstrate that geriatric patients and non-geriatric patients have similar rates of mortalities and lengths of stay in hospital even though the rate of complication was significantly higher in the older group. This is in indication that with the appropriate geriatric trauma care older severely injured people still have very favourable outcomes even compared to younger patients. Therefore, we think there is clearly a need to implement more dedicated geriatric trauma teams/centres to meet the specific needs of the injured geriatric patient. Furthermore, each geriatric patient must be assessed and evaluated individually, as chronologic age might not necessarily correlate with biological age.

## Figures and Tables

**Table 1:** Baseline characteristics and comparison between the age groups.

		Age < 70				Age ≥ 70			
		*n*	%	Mean	Standard Deviation	*n*	%	Mean	Standard Deviation
gender	female	276	24.8%			159	46.0%		
	male	835	75.2%			187	54.0%		
trauma mechanism	penetrating	68	6.1%			3	0.9%		
	blunt	1043	93.9%			343	99.1%		
ISS (points)				31	15			37	22
**At admission**									
heart rate (beats/min)				91	23			85	21
systolic blood pressure (mmHg)				125	26			133	29
base excess (mmol/L)				−4.1	4.8			−3.6	4.4
lactate (mmol/L)				2.5	2.3			2.2	2.1
haemoglobin (g/L)				11.43	2.60			10.93	2.22
haematokrit (L/L)				33.26	7.30			32.15	6.12
thrombocytes (10^3^/µL)				204	72			186	77
GCS (points)				10	5			9	5
presence of coagulopathy (INR > 1.4)		237	21.3%			61	30.2%		
**Outcome**									
death during hospitalisation		179	16.1%			104	46.0%		
Length of stay (days)				17	19			10	11
complications		593	53.4%			158	45.7%		

ISS: Injury Severity Score; GCS: Glasgow coma scale; INR: international normalized ratio.

**Table 2:** Matching results and base characteristics of the matched couples.

Group	Male (%)	Age (Years ± SD)	ISS (Points ± SD)	GCS (Points ± SD)	BE (mmol/L ± SD)	INR ≥ 1.4 (%)
*n* < 70 yo (*n* = 57)	72	45 ± 16	33 ± 22	10 ± 6	−2.6 ± 2.5	8.9
*n* ≥ 70 yo (*n* = 57)	72	79 ± 6	33 ± 22	10 ± 6	−2.6 ± 2.7	8.9

SD: standard deviation; ISS: Injury Severity Score; GCS: Glasgow coma scale; BE: base excess; INR: International standardized ratio; yo: years old.
